# Physicochemical and Antibacterial Properties of Bioactive Retrograde Filling Materials

**DOI:** 10.3390/bioengineering9110624

**Published:** 2022-10-28

**Authors:** Tarek Ashi, Davide Mancino, Louis Hardan, Rim Bourgi, Jihed Zghal, Valentina Macaluso, Sharif Al-Ashkar, Sleman Alkhouri, Youssef Haikel, Naji Kharouf

**Affiliations:** 1 Department of Biomaterials and Bioengineering, INSERM UMR_S 1121, Biomaterials and Bioengineering, 67000 Strasbourg, France; 2 Department of Endodontics, Faculty of Dental Medicine, Strasbourg University, 67000 Strasbourg, France; 3 Pôle de Médecine et Chirurgie Bucco-Dentaire, Hôpital Civil, Hôpitaux Universitaire de Strasbourg, 67000 Strasbourg, France; 4 Department of Restorative Dentistry, School of Dentistry, Saint-Joseph University, Beirut 1107 2180, Lebanon; 5 Laboratoire Energetique Mecanique Electromagnetisme, University of Paris Ouest, 50 Rue de Sèvres, 92410 Ville d’Avray, France; 6 ICube Laboratory, UMR 7357 CNRS, Mechanics Department, University of Strasbourg, 67000 Strasbourg, France; 7 ESTA, School of Business & Technology, 90000 Belfort, France; 8 Faculty of Dentistry, Al Sham Private University (ASPU), Damascus 0100, Syria; 9 Division Regenerative Orofacial Medicine, Department of Oral and Maxillofacial Surgery, University Medical Center Hamburg-Eppendorf, 20246 Hamburg, Germany

**Keywords:** calcium silicate cement, retrograde materials, premixed cement, powder–liquid cement

## Abstract

The purpose of the present study was to evaluate the physicochemical properties and antibacterial activity of three calcium silicate cements. Mineral trioxide aggregate (MTA Biorep “BR”), Biodentine (BD) and Well-Root PT (WR) materials were investigated using scanning electron microscopy (SEM) at 24, 72 and 168 h of immersion in phosphate buffered saline (PBS). The antibacterial activity against *Enterococcus faecalis* (*E. faecalis*), the solubility, roughness, pH changes and water contact angle were also analyzed. All results were statistically analyzed using a one-way analysis of variance test. Statistically significant lower pH was detected for BD than WR and BR (*p* < 0.05). No statistical difference was found among the three materials for the efficacy of kill against *E. faecalis* (*p* > 0.05). Good antibacterial activity was observed (kill 50% of *bacteria*) after 24 h of contact. The wettability and the roughness of BR were higher than for the other cements (*p* < 0.05). BD was more soluble than WR and BR (*p* < 0.05). In conclusion, the use of bioceramic cements as retrograde materials may play an important role in controlling bacterial growth and in the development of calcium phosphate surface layer to support healing. Moreover, the premixed cement was easier to use than powder–liquid cement.

## 1. Introduction

The success of surgical endodontic treatment requires root-end filling materials that are easy to use, biocompatible, stable and economical [1,2,3]. The goal is to seal the apex hermetically and prevent microorganisms from entering the root canal [4,5].

Retrograde root-end filling materials have included zinc oxide eugenol cements, amalgam, glass ionomer and resins which have failed to meet the ideal requirements of root-end filling treatment [6,7].

Calcium silicate cement materials, colloquially denoted as “Bioceramic”, in both forms, sealer [8] or thicker mixture [9], are considered as the ideal endodontic material for retrograde treatment due to their excellent physicochemical and biological properties [10,11,12,13,14,15], including biocompatibility and stability. These inorganic and non-corrosive ceramic cements contain tricalciums silicate and various radiopaque powders [16].

Mineral Trioxide aggregate (MTA) was the original calcium silicate cement introduced for endodontic treatment in 1993 and it is considered as the gold-standard material for various endodontic applications [10]. Other tricalcium silicate-based products have been developed, improvements on the original Portland cement invention [10]. MTA Biorep (Itena Clinical, Paris, France) is a powder–liquid product containing calcium silicate cement and calcium tungstate. Its water-based liquid, containing an organic plasticizer, improves the handling and plasticity [10].

Biodentine™ (Septodont, Saint-Maur-des-fossés, France) is a calcium silicate cement material and it has higher strength than other similar products [17]. This product consists of a powder–liquid material, where the liquid contains calcium chloride with an admixture of polycarboxylate [17]. MTA Biorep and Biodentine cements are indicated for several endodontic treatments, including pulpotomy, pulp capping, resorption, apicoectomy and open apex [9,10,18,19,20,21].

Some bioceramic cements require manual mixing and handling, powder–liquid systems, which require certain skills [16,22]. In addition, any change in the powder–liquid ratio or the mixing could affect and alter the physicochemical properties of these cements [8,23]. Premixed cements have been introduced to avoid errors during manual mixing. These premixed materials do not require any preparation before clinical application [8,16]. As mentioned in previous studies [8,16], these premixed materials are advantageous for some clinicians in the handling.

Well-Root™ PT is a novel premixed calcium aluminosilicate cement delivered in capsules for direct clinical use [24]. No information was found in the literature on the antibacterial activity and the physicochemical properties of this cement.

The purpose of the present research was to investigate the physicochemical properties and the antibacterial activity of three calcium silicate cements. The hypothesis concerned whether there would be antibacterial and physicochemical differences between the three tested materials.

## 2. Materials and Methods

### 2.1. Materials

MTA Biorep “BR” (Itena Clinical, Paris, France), Biodentine™ “BD” (Septodont, Saint-Maur-des-fossés, France) and Well-Root™ PT “WR” (Vericom, Gangwon-Do, Korea) were used in the present study, following the manufacturer’s instructions (Table 1). All specimens were conserved in the dark in a container at 37 °C and 95% relative humidity for 48 h until completely set [25].

### 2.2. pH Measurements of the Aqueous Solution in Contact with the Cement

Five samples of each group were prepared using Teflon molds (3.8 mm in high and 3 mm in diameter). Each sample was put in contact with 10 mL distilled water at 37 °C. A pH meter, “CyberScan pH 510” (Thermo Scientific, Waltham, Massachusetts, USA), was used to measure the pH of water at 3, 24, 72 and 168 h. Before each pH test, the calibration of pH meter was performed using standard solutions at pH 10, 4 and 7 (Hanna Instruments, Lingolsheim, France). Distilled water was used to rinse and eliminate the previous solution from the pH meter electrode.

### 2.3. Solubility

Five samples (2 mm in height and 20 mm in diameter) of each material were analyzed following the method of a previous study [26]. The samples were weighed using a digital system, then the disks were immersed for 24 h in 50 mL of water at 37 °C. The samples were removed from distilled water and then dried at 37 °C for 24 h. Finally, the samples were weighed again to obtain the final weight. The solubility was defined from the difference in mass between the final and the initial weight.

### 2.4. Scanning Electron Microscope (SEM) of Crystallites Creation

Twelve samples for each material were created (3.8 mm in high and 3 mm in diameter. After the setting time, as described in Section 2.1, three samples from each group were stored in hermetic boxes and kept in dry condition. The remaining samples (9 samples) from each group were put in 10 mL of phosphate-buffered saline (PBS10×, Dominique Dutscher, Bernolsheim, France) at 37 °C. After 24, 72 and 168 h in PBS, 3 samples for each period were washed with distilled water for 5 min, sputter-coated with gold–palladium (20/80) [27], then, analyzed using an SEM (FEI Company, Eindhoven, The Netherlands, 10 kV) at a magnification of 5000×. Energy Dispersive X-ray (EDX) analysis was used during an acquisition time of 1 min and a working length of 10 mm to attain the spectrum of chemical elements present on the surface.

### 2.5. Roughness and Water Sorption Tests

Five samples from each product were created using Teflon molds (10 mm in diameter and 2 mm in height). After the setting time, as described in Section 2.1, the samples were kept in dry in the fume hood overnight. The roughness of each surface was measured using a 3D digital profilometer (Keyence, Osaka, Japan) at 2500× magnification. The average roughness (Sa) was calculated using software (Keyence 7000 VHX, Osaka, Japan).

After measuring the surface roughness, on the same samples, a contact angle device (Biolin Scientific, Espoo, Finland) was used to observe the infiltration time of a 5 µL droplet of water into the material surface. A movie was recorded to track the profile and the absorption time of the water droplet.

### 2.6. Antimicrobial Activity

Brain Heart Infusion medium (BHI) (Darmstadt, Germany) was used to culture *Enterococcus faecalis* (*E. faecalis*, ATCC 29212). The turbidity was adjusted to OD_600_ (nm) = 0.3. A direct contact test (DCT) was performed to investigate the antibacterial activity of the three products against *E. faecalis*. Triplicate samples were placed in 24-well culture plates. One milliliter of the bacterial medium was put to each well and incubated anaerobically for 24 h at 37 °C (constant stirring at 450 rpm). The bacterial medium without the cement materials was used as the control group. After 24 h, 10-fold serial dilutions up to 10^6^ in BHI were performed on each specimen. One hundred microliters of each diluted medium was added onto a BHI agar plate, homogeneously spread and incubated at 37 °C for 24 h. Manual CFU/mL (colony forming units/mL) counting was measured the *E. faecalis* concentration.

### 2.7. Statistical Analysis

The results of pH, solubility, roughness and antibacterial activity were statistically analyzed using the Kruskal–Wallis test along with the Tukey Test. SigmaPlot release 11.2 (Systat Software, Inc., San Jose, CA, USA) was used with a statistical significance was set at α = 0.05.

## 3. Results

### 3.1. pH Measurements

**Figure 1:**
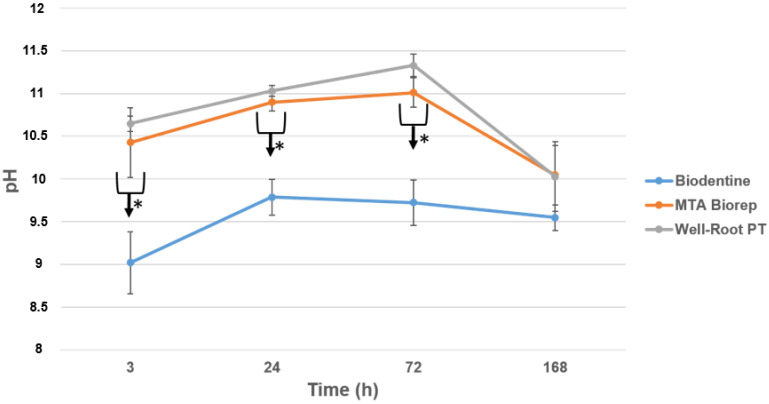
pH changes for the three products after 3, 24, 72 and 168 h of contact with water. * *p* < 0.05.

The pH of the solution in contact with the three cements over 7 days is shown in Figure 1. All three cements were alkaline for the solution for up to 72 h. BR and WR demonstrated statistically higher pH than BD at all time points (3, 24, 72 and 168 h) (*p* < 0.05). No significance difference was found between BR and WR (*p* > 0.05).

### 3.2. Solubility

**Figure 2:**
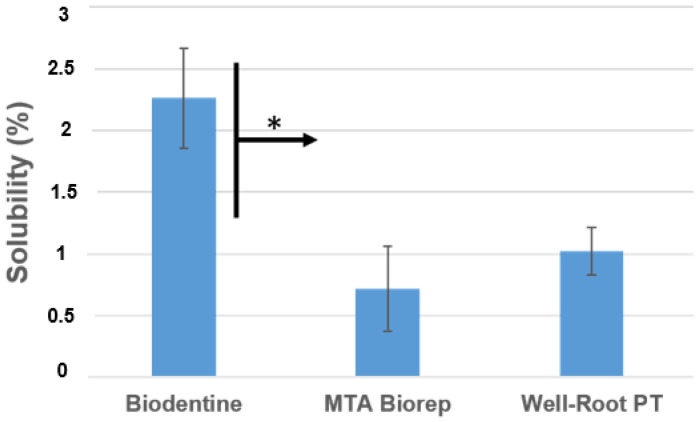
Solubility percentages of the different products after aging in water for 24 h at 37 °C. * *p* < 0.05.

The mean and standard deviation of solubility (wt.%) values are presented in Figure 2. BD was more soluble than BR and WR at 24 h (*p* < 0.05).

### 3.3. Scanning Electron Microscope (SEM)

**Figure 3:**
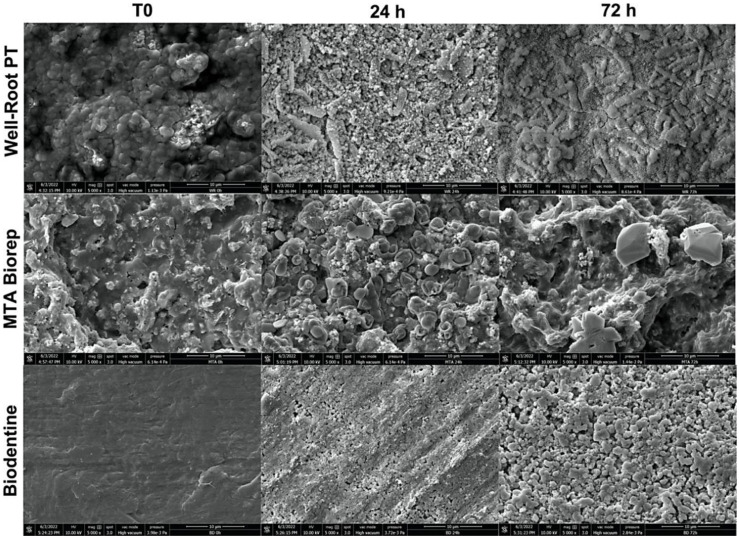
Scanning electron microscope images (5000× magnification) demonstrate the morphological changes of each material at 24 and 72 h of immersion in phosphate-buffered solution at 37 °C.

**Figure 4:**
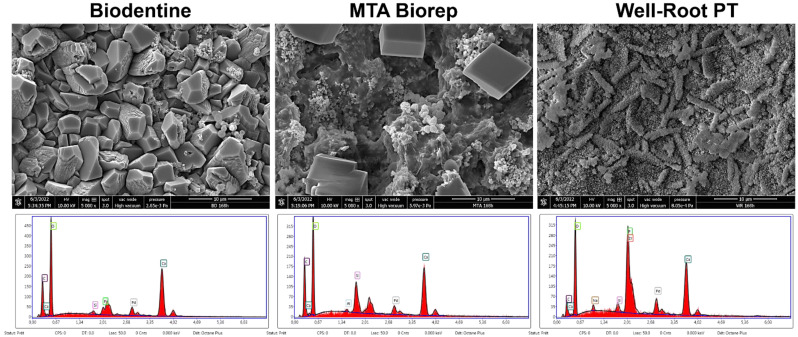
Scanning electron microscope images (5000× magnification) demonstrate the morphological changes and EDX spectrums of each material at 168 h of immersion in phosphate-buffered solution at 37 °C.

The crystalline structures of the three cements are shown in Figure 3 and Figure 4. All three cements had crystalline deposits after immersion in PBS at 37 °C. At each immersion period (24, 72 and 168 h), different crystalline appearances were observed. At 24 and 72 h, WR had elongated crystals, BD and BR had globular and cubic crystals (Figure 3).

After 168 h, BR and BD showed cubic crystals. The cubic crystals of BR were larger (8–10 µm) than for BD (3–6 µm). WR had globular and elongated crystal features at 168 h. EDX analysis for the three cements after 168 h in PBS showed different percentages of Ca, P and Si among the three materials. Other chemical elements were detected on WR (Zr) and BR (Al) surfaces.

### 3.4. Roughness and Water Sorption Tests

**Figure 5:**
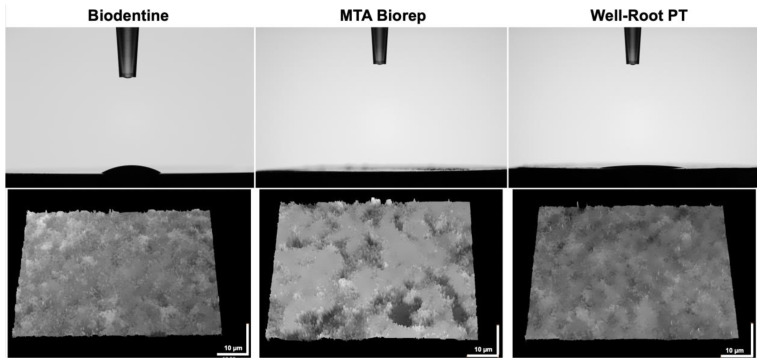
Contact angles of 5 µL of water drop on the different cement surfaces after 10 s. Digital micrographs of the different surfaces using KEYENCE 7000 VHX demonstrate the roughness of each material.

BR demonstrated the highest hydrophilicity for a 5 µL of a drop of distilled water compared to BD and WR. Contact angles of 15° and 9° for BD and WR, respectively, were investigated after 10 s (Table 2). Whereas, the water sorption in the BR surface was faster (<10 s) than the other cement surfaces. The contact angle of the drop in contact with BR surface after 10 s was 0° (Figure 5). All tested cement surfaces were analyzed using KEYENCE 7000 VHX to measure the roughness of these surfaces. In addition, rougher surfaces were obtained for BR and BD compared with WR surfaces (*p* < 0.05) (Table 2 and Figure 5).

### 3.5. Antimicrobial Activity

**Figure 6:**
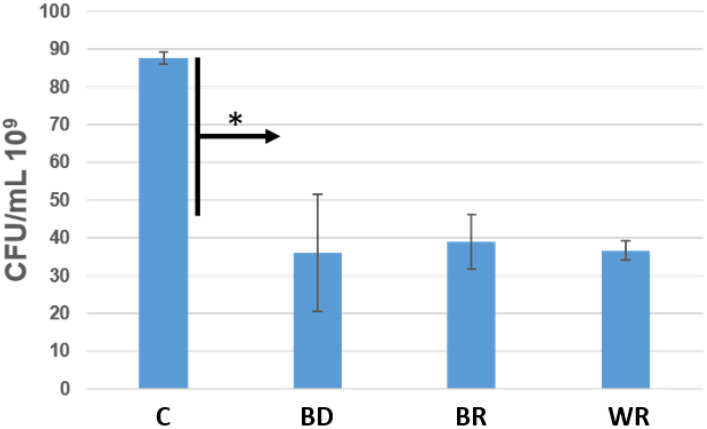
Number of colony-forming units/mL of *Enterococcus faecalis* in contact with Biodentine (BD), MTA Biorep (BR) and Well-Root PT (WR) after 24 h at 37 °C in anaerobic conditions. * *p* < 0.05.

Bacterial growth was significantly inhibited with the three cements. No significant difference was found among them for the efficiency against *E.faecalis* (*p* > 0.05). The three cements killed about 50% of the *bacteria* after 24 h, versus the control (*p* < 0.05) (Figure 6).

## 4. Discussion

Since their introduction in the dental market, calcium silicate cement materials have attained popularity due to their excellent physicochemical, biological and mechanical properties and their positive outcomes in clinical applications [9,28]. Calcium silicate cement products are the ideal dentine repair materials for various endodontic applications [2,3,17,20,29]. A number of investigations have been conducted to determine the differences among the products as retrograde bioactive material.

Our present in vitro study comparing BR, BD and WR showed significant antibacterial activity and formation of crystals on their surfaces after immersion in PBS. Therefore, the null hypothesis was partially rejected.

Alkaline pH was detected with the three materials (Figure 1), but BD had a lower pH than WR and BR (*p* < 0.05). The alkalinity is key to the antibacterial activity and healing process [8,30,31,32]. Kharouf et al. [10] measured a high alkaline pH with MTA Biorep. Oliveira et al. [33] measured a lower pH for BD (pH = around 6–7) after 24 h than the one attained our study (pH = around 9–10), whilst Hassan et al. [34] measured higher pH values (pH = around 11–12) for BD after 24 h. The differences may be related to the methods of exposing the materials. The premixed bioceramic cement (WR) created a similar pH to the powder–liquid BR cement.

In the present study, the solubility of the three cements did not exceed the 3% mass after 24 h in distilled water; however, the ISO 6876 was not used. The results of the present study agree with those of the Al-Sherbiny study [35] for BD results and with the study of Queiroz et al. [36] for MTA Repair HP. The premixed bioceramic (WR) had a solubility similar to that of the other two cements (Figure 2). Solubility is important because if it is high, voids and gaps may be formed, which would be a pathway for the microorganisms to re-infect the root canal system [8,27]. BD demonstrated lower pH values than the other products, but the solubility of BD was higher. Weckwerth et al. [37] noted that a higher solubility does not always correlate with higher pH. The cement may release other components, which do not have any effect on pH changes and the liberation of these components increases the solubility of this material.

The direct contact test was used in this in vitro study to evaluate the antibacterial activity of the different cements. The agar contact test was not used, because in our previous study [8], we noted that these cements infiltrate the agar plates and hide the inhibition zones. *E. faecalis* was used in our experiment because this Gram-positive facultative anaerobe microorganism is the most predominant *bacterium* found in root canal infections and failure [8,38,39,40,41]. No significant differences were found among the capacity of killing bacteria of the three cements (*p* > 0.05). All the materials demonstrated high potential of killing bacteria after 24 h (kill around 50%) compared to the control group (bacterial medium). The antibacterial activity of these cements comes from the high alkaline pH [8,10,16,42,43].

All the three cements had different crystalline features (Figure 3 and Figure 4) after immersion in PBS. Cubic crystals were observed on BR and BD samples after 7 d of immersion in PBS (Figure 4). The crystallites of BD were more numerous and smaller than BR crystallites. Elongated crystals were observed on WR surfaces. Yoo et al. [44] showed the importance of biomineralization to entomb the microorganisms in dentinal tubules, since the elimination of 100% of bacteria from the root canal system is impossible [45,46]. EDX analysis showed different chemical compositions of formed crystals onto each cement. Ca, Si and P were detected on the surfaces of the three materials, which reflects the reactions between calcium silicate and PBS. Zr presented onto WR and Al onto BR surface due to the initial composition which contain Zirconium oxide and Tricalcium aluminate, respectively. Al was not detected on WR surface which is a calcium aluminosilicate compound. Therefore, EDX could be considered as a qualitative method and the composition of these crystals could not be identified without X-ray diffraction analysis, which could be considered as limitation of this in vitro study.

A contact angle test was used to determine the capacity of absorption of 5 µL drops of distilled water. This test is an indicator of the wetting behavior of a solid material (cement) and a liquid (water). Contact angle measurement is affected by the surface roughness [47] and the chemical surface composition [48]. The roughnesss of WR surface was less than that of BR and BD (*p* < 0.05), which could be related to the particles size of each cement. After 10 s, the 5 µL drop was totally absorbed by the surface of BR which had the higher roughness values compared to BD and WR (*p* < 0.05). Whatever the considered wetting model (Wenzel or Cassie–Baxter) [48], the higher roughness and hydrophilic surface would increase the adhesion, protein adsorption and the cellular attachment, and provide a superior biocompatibility [48,49,50]. In contrast, a decrease in cell proliferation and growth could be related to a critical roughness ration, where the elastic energy of the cell hinders the insertion of the cells into surface trenches, where cells install over the tips of the rough surfaces leading to only point-contact, which minimizes cell–surface interaction [51].

Further studies are required to investigate the cytotoxicity, the setting time, the flowability, calcium ions releasing and the filling ability of the novel premixed cement.

## 5. Conclusions

Within the limitations of the present study, the three calcium silicate cement products, MTA Biorep, Biodentine and Well-Root PT, had a high antibacterial activity, formation of phosphate crystal in PBS alkaline and had comparable solubility. The premixed format was more convenient as a retrograde agent.

## Figures and Tables

**Table 1:** Manufacturer and manipulation of the tested materials.

Materials	Manufacturer	Lot	Mixing	Composition
MTA Biorep	Itena Clinical, Paris, France	53505	Powder: 1 capsule Liquid: 4 drops	Powder: Tricalcium silicate; Dicalcium silicate; Tricalcium aluminate; Calcium oxide; Calcium Tungstate Liquid: Water and Plasticizer.
Biodentine™	Septodont, Saint-Maur-des-fossés, France	B28033	Powder: 1 capsule Liquid: 5 drops	Powder: Tricalcium silicate; Dicalcium silicate; Calcium carbonate; Zirconiom dioxide; Iron oxide Liquid: Calcium chloride; Hydrosoluble polymer
Well-Root™ PT	Vericom, Gangwon-Do, Korea	WT010100	Premixed	Calcium aluminosilicate compound; Zirconium oxide; Thickening agent

**Table 2:** Contact angles of 5 µL of distilled water on the different material surfaces after 10 s of deposition. Mean and standard deviations of the roughness (Sa) of the tested materials. Superscript letters a, b, c and x, y, z indicate statistical significance (*p* < 0.05).

Test\Materials	Biodentine	MTA Biorep	Well-Root PT	Statistical Significance
Contact angle (°)	15.2 ± 3.5 ^x^	0 ^y^	8.9 ± 0.4 ^z^	*p* < 0.05
Roughness (Sa)	0.7 ± 0.05 ^a^	0.9 ± 0.2 ^b^	0.3 ± 0.02 ^c^	*p* < 0.05

## Data Availability

The data presented in this study are available on request from the corresponding author.

## References

[1] Murata K., Washio A., Morotomi T., Rojasawasthien T., Kokabu S., Kitamura C. (2021). Physicochemical Properties, Cytocompatibility, and Biocompatibility of a Bioactive Glass Based Retrograde Filling Material. Nanomaterials.

[2] Parirokh M., Torabinejad M. (2010). Mineral Trioxide Aggregate: A comprehensive literature review—Part III: Clinical applications, drawbacks, and mechanism of action. J. Endod..

[3] Roberts H.W., Toth J.M., Berzins D.W., Charlton D.G. (2008). Mineral trioxide aggregate material use in endodontic treatment: A review of the literature. Dent. Mater..

[4] Cohen S., Burns R. (1994). Pathways of the Pulp.

[5] Ingle J.I., Bakland L.K., Baumgartner J.C. (2008). Ingle’s Endodontics 6.

[6] Viswanath G., Tilakchand M., Naik B.D., Kalabhavi A.S., Kulkarni R.D. (2021). Comparative evaluation of antimicrobial and antifungal efficacy of bioactive root-end filling materials: An in vitro study. J. Conserv. Dent..

[7] Suhag A., Chhikara N., Pillania A., Yadav P. (2018). Root end filling materials: A review. Indian J. Dent. Sci..

[8] Kharouf N., Arntz Y., Eid A., Zghal J., Sauro S., Haikel Y., Mancino D. (2020). Physicochemical and Antibacterial Properties of Novel, Premixed Calcium Silicate-Based Sealer Compared to Powder–Liquid Bioceramic Sealer. J. Clin. Med..

[9] Eid A., Mancino D., Rekab M.S., Haikel Y., Kharouf N. (2022). Effectiveness of Three Agents in Pulpotomy Treatment of Permanent Molars with Incomplete Root Development: A Randomized Controlled Trial. Healthcare.

[10] Kharouf N., Zghal J., Addiego F., Gabelout M., Jmal H., Haïkel Y., Bahlouli N., Ball V. (2021). Tannic Acid Speeds up the Setting of Mineral Trioxide Aggregate Cements and Improves Its Surface and Bulk Properties. J. Colloid Interface Sci..

[11] Hardan L., Mancino D., Bourgi R., Alvarado-Orozco A., Rodríguez-Vilchis L.E., Flores-Ledesma A., Cuevas-Suárez C.E., Lukomska-Szymanska M., Eid A., Danhache M.-L. (2022). Bond Strength of Adhesive Systems to Calcium Silicate-Based Materials: A Systematic Review and Meta-Analysis of In Vitro Studies. Gels.

[12] Kang T.-Y., Choi J.-W., Seo K.-J., Kim K.-M., Kwon J.-S. (2021). Physical, Chemical, Mechanical, and Biological Properties of Four Different Commercial Root-End Filling Materials: A Comparative Study. Materials.

[13] Vergaças J.H.N., de Lima C.O., Barbosa A.F.A., Vieira V.T.L., Dos Santos Antunes H., da Silva E.J.N.L. (2022). Marginal gaps and voids of three root-end filling materials: A microcomputed tomographic study. Microsc. Res. Tech..

[14] Džanković A., Hadžiabdić N., Korać S., Tahmiščija I., Konjhodžić A., Hasić-Branković L. (2020). Sealing Ability of Mineral Trioxide Aggregate, Biodentine and Glass Ionomer as Root-End Materials: A Question of Choice. Acta Med. Acad..

[15] Jardine A.P., Rosa K.F.V., Matoso F.B., Quintana R.M., Grazziotin-Soares R., Kopper P.M.P. (2021). Marginal gaps and internal voids after root-end filling using three calcium silicate-based materials: A Micro-CT analysis. Braz. Dent. J..

[16] Debelian G., Trope M. (2016). The use of premixed bioceramic materials in endodontics. G. Ital. Di Endod..

[17] Nowicka A., Lipski M., Parafiniuk M., Sporniak-Tutak K., Lichota D., Kosierkiewicz A., Kaczmarek W., Buczkowska-Radlińska J. (2013). Response of human dental pulp capped with biodentine and mineral trioxide aggregate. J. Endod..

[18] Taha N.A., Al-Rawash M.H., Imran Z.A. (2022). Outcome of full pulpotomy in mature permanent molars using 3 calcium silicate-based materials: A parallel, double blind, randomized controlled trial. Int. Endod. J..

[19] Jovanović L.Z., Bajkin B.V. (2021). Scanning electron microscopy analysis of marginal adaptation of mineral trioxide aggregate, tricalcium silicate cement, and dental amalgam as a root end filling materials. Microsc. Res. Tech..

[20] Selvendran K.E., Ahamed A.S., Krishnamurthy M., Kumar V.N., Raju V.G. (2022). Comparison of three different materials used for indirect pulp capping in permanent molars: An in vivo study. J. Conserv. Dent..

[21] Sanaei-Rad P., Bolbolian M., Nouri F., Momeni E. (2021). Management of internal root resorption in the maxillary central incisor with fractured root using Biodentine. Clin. Case Rep..

[22] Motwani N., Ikhar A., Nikhade P., Chandak M., Rathi S., Dugar M., Rajnekar R. (2021). Premixed bioceramics: A novel pulp capping agent. J. Conserv. Dent..

[23] Cavenago B.C., Pereira T.C., Duarte M.A.H., Ordinola-Zapata R., Marciano M.A., Bramante C.M., Bernardineli N. (2014). Influence of powder-to-water ratio on radiopacity, setting time, pH, calcium ion release and a micro-CT volumetric solubility of white mineral trioxide aggregate. Int. Endod. J..

[24] Jeon J., Choi N., Kim S. (2021). Color Change in Tooth Induced by Various Calcium Silicate-Based Pulp-Capping Materials. J. Korean Acad. Pediatr. Dent..

[25] Kharouf N., Sauro S., Hardan L., Fawzi A., Suhanda I.E., Zghal J., Addiego F., Affolter-Zbaraszczuk C., Arntz Y., Ball V. (2022). Impacts of Resveratrol and Pyrogallol on Physicochemical, Mechanical and Biological Properties of Epoxy-Resin Sealers. Bioengineering.

[26] Kharouf N., Mancino D., Zghal J., Helle S., Jmal H., Lenertz M., Viart N., Bahlouli N., Meyer F., Haikel Y. (2021). Dual role of Tannic acid and pyrogallol incorporated in plaster of Paris: Morphology modification and release for antimicrobial properties. Mater. Sci. Eng. C Mater. Biol. Appl..

[27] Kharouf N., Sauro S., Jmal H., Eid A., Karrout M., Bahlouli N., Haikel Y., Mancino D. (2021). Does Multi-Fiber-Reinforced Composite-Post Influence the Filling Ability and the Bond Strength in Root Canal?. Bioengineering.

[28] Camilleri J. (2020). Classification of hydraulic cements used in dentistry. Front. Dent. Med..

[29] Kaur M., Singh H., Dhillon J.S., Batra M., Saini M. (2017). MTA versus Biodentine: Review of Literature with a Comparative Analysis. J. Clin. Diagn. Res..

[30] Drukteinis S., Peciuliene V., Shemesh H., Tusas P., Bendinskaite R. (2019). Porosity Distribution in Apically Perforated Curved Root Canals Filled with Two Different Calcium Silicate Based Materials and Techniques: A Micro-Computed Tomography Study. Materials.

[31] Urban K., Neuhaus J., Donnermeyer D., Schäfer E., Dammaschke T. (2018). Solubility and pH Value of 3 Different Root Canal Sealers: A Long-term Investigation. J. Endod..

[32] Poggio C., Dagna A., Ceci M., Meravini M.-V., Colombo M., Pietrocola G. (2017). Solubility and pH of bioceramic root canal sealers: A comparative study. J. Clin. Exp. Dent..

[33] Oliveira L.V., de Souza G.L., da Silva G.R., Magalhães T.E.A., Freitas G.A.N., Turrioni A.P., de Rezende Barbosa G.L., Moura C.C.G. (2021). Biological parameters, discolouration and radiopacity of calcium silicate-based materials in a simulated model of partial pulpotomy. Int. Endod. J..

[34] Hassan T., Zeid A., Alothmani O.S., Yousef M. (2015). Biodentine and Mineral Trioxide Aggregate: An Analysis of Solubility, pH Changes and Leaching Elements. Life Sci. J..

[35] Al-Sherbiny I.M., Farid M.H., Abu-Seida A.M., Motawea I.T., Bastawy H.A. (2021). Chemico-physical and mechanical evaluation of three calcium silicate-based pulp capping materials. Saudi Dent. J..

[36] Queiroz M.B., Torres F.F.E., Rodrigues E.M., Viola K.S., Bosso-Martelo R., Chavez-Andrade G.M., Guerreiro-Tanomaru J.M., Tanomaru-Filho M. (2021). Physicochemical, biological, and antibacterial evaluation of tricalcium silicate-based reparative cements with different radiopacifiers. Dent. Mater..

[37] Weckwerth P.H., Machado A.C., Kuga M.C., Vivan R.R., Polleto Rda S., Duarte M.A. (2012). Influence of radiopacifying agents on the solubility, pH and antimicrobial activity of portland cement. Braz. Dent. J..

[38] Alsubait S., Albader S., Alajlan N., Alkhunaini N., Niazy A., Almahdy A. (2019). Comparison of the antibacterial activity of calcium silicate- and epoxy resin-based endodontic sealers against Enterococcus faecalis biofilms: A confocal laser-scanning microscopy analysis. Odontology.

[39] Chang S.-W., Lee S.-Y., Kang S.-K., Kum K.-Y., Kim E.-C. (2014). In vitro biocompatibility, inflammatory response, and osteogenic potential of 4 root canal sealers: Sealapex, Sankin apatite root sealer, MTA Fillapex, and iRoot SP root canal sealer. J. Endod..

[40] Portela C.A., Smart K.F., Tumanov S., Cook G.M., Villas-Bôas S.G. (2014). Global metabolic response of Enterococcus faecalis to oxygen. J. Bacteriol..

[41] Stuart C.H., Schwartz S.A., Beeson T.J., Owatz C.B. (2006). Enterococcus faecalis: Its role in root canal treatment failure and current concepts in retreatment. J. Endod..

[42] López-García S., Myong-Hyun B., Lozano A., García-Bernal D., Forner L., Llena C., Guerrero-Gironés J., Murcia L., Rodríguez-Lozano F.J. (2020). Cytocompatibility, bioactivity potential, and ion release of three premixed calcium silicate-based sealers. Clin. Oral. Investig..

[43] Camilleri J. (2011). Characterization and hydration kinetics of tricalcium silicate cement for use as a dental biomaterial. Dent. Mater..

[44] Yoo J.S., Chang S.W., Oh S.R., Perinpanayagam H., Lim S.M., Yoo Y.J., Oh Y.R., Woo S.B., Han S.H., Zhu Q. (2014). Bacterial entombment by intratubular mineralization following orthograde mineral trioxide aggregate obturation: A scanning electron microscopy study. Int. J. Oral. Sci..

[45] Mancino D., Kharouf N., Cabiddu M., Bukiet F., Haïkel Y. (2021). Microscopic and chemical evaluation of the filling quality of five obturation techniques in oval-shaped root canals. Clin. Oral Investig..

[46] Mancino D., Kharouf N., Hemmerlé J., Haïkel Y. (2019). Microscopic and Chemical Assessments of the Filling Ability in Oval-Shaped Root Canals Using Two Different Carrier-Based Filling Techniques. Eur. J. Dent..

[47] Kontakiotis E.G., Tzanetakis G.N., Loizides A.L. (2007). A comparative study of contact angles of four different root canal sealers. J. Endod..

[48] Ball V. (2018). Self-Assembly Processes at Interfaces.

[49] Colombo M., Poggio C., Dagna A., Meravini M.-V., Riva P., Trovati F., Pietrocola G. (2018). Biological and physico-chemical properties of new root canal sealers. J. Clin. Exp. Dent..

[50] Hachem C.E., Chedid J.C.A., Nehme W., Kaloustian M.K., Ghosn N., Sahnouni H., Mancino D., Haikel Y., Kharouf N. (2022). Physicochemical and Antibacterial Properties of Conventional and Two Premixed Root Canal Filling Materials in Primary Teeth. J. Funct. Biomater..

[51] Majhy B., Priyadarshini P., Sen A.K. (2021). Effect of surface energy and roughness on cell adhesion and growth—facile surface modification for enhanced cell culture. RSC Adv..

